# Phosphatidylserine synthase regulates cellular homeostasis through distinct metabolic mechanisms

**DOI:** 10.1371/journal.pgen.1008548

**Published:** 2019-12-23

**Authors:** Xiao Yang, Jingjing Liang, Long Ding, Xia Li, Sin-Man Lam, Guanghou Shui, Mei Ding, Xun Huang

**Affiliations:** 1 State Key Laboratory of Molecular Developmental Biology, Institute of Genetics and Developmental Biology, Chinese Academy of Sciences, Beijing, China; 2 University of Chinese Academy of Sciences, Beijing, China; 3 School of Life Sciences, Shandong First Medical University & Shandong Academy of Medical Sciences, TaiAn, China; 4 LipidAll Technologies Co., Ltd. Changzhou, China; German Cancer Research Center (DKFZ), GERMANY

## Abstract

Phosphatidylserine (PS), synthesized in the endoplasmic reticulum (ER) by phosphatidylserine synthase (PSS), is transported to the plasma membrane (PM) and mitochondria through distinct routes. The *in vivo* functions of PS at different subcellular locations and the coordination between different PS transport routes are not fully understood. Here, we report that *Drosophila* PSS regulates cell growth, lipid storage and mitochondrial function. In *pss RNAi*, reduced PS depletes plasma membrane Akt, contributing to cell growth defects; the metabolic shift from phospholipid synthesis to neutral lipid synthesis results in ectopic lipid accumulation; and the reduction of mitochondrial PS impairs mitochondrial protein import and mitochondrial integrity. Importantly, reducing PS transport from the ER to PM by loss of *PI4KIIIα* partially rescues the mitochondrial defects of *pss RNAi*. Together, our results uncover a balance between different PS transport routes and reveal that PSS regulates cellular homeostasis through distinct metabolic mechanisms.

## Author summary

Phosphatidylserine (PS), a membrane phospholipid synthesized in the endoplasmic reticulum (ER) by the enzyme phosphatidylserine synthase (PSS), is transported to the plasma membrane (PM) and mitochondria through different paths. The cellular functions of PS at different places in the cell and the mechanisms that coordinate the different PS transport paths are not fully understood. Here, we identified that PSS regulates cell growth, lipid storage and mitochondrial function in the fruit fly larval salivary gland. We showed that loss of *pss* function has three effects: (1) reduced levels of PS lead to reduced levels of plasma membrane Akt, a key component in the insulin pathway, which is important for cell growth; (2) it causes a shift from phospholipid synthesis to neutral lipid synthesis, which results in excess lipid accumulation; and (3) it reduces the level of mitochondrial PS, which impairs mitochondrial protein import and mitochondrial morphology. We also found that reducing the transport of PS from the ER to PM partially rescues the mitochondrial defects caused by loss of *pss* function. Together, our results reveal that PSS regulates cellular homeostasis through distinct metabolic changes, and uncover a balance between different PS transport pathways.

## Introduction

Phospholipids make up the membranes that separate cells from extracellular environments and enclose subcellular compartments. Besides their structural role in membranes, phospholipids and their modification products also have specific intracellular and/or intercellular roles in many cellular processes [1]. The synthesis and the subcellular distribution of phospholipids are important for their function.

Phosphatidylserine (PS) is synthesized in regions of the endoplasmic reticulum (ER) called MAMs (mitochondria-associated membranes) [2, 3], and is then imported into mitochondria for phosphatidylethanolamine (PE) synthesis by mitochondrial-localized phosphatidylserine decarboxylase [4], or transported to the plasma membrane (PM) [5, 6]. In the PM, PS mainly resides in the inner leaflet, and loss of this asymmetry acts as an “eat me” signal to trigger apoptotic cell death [7].

In mammals, PSS1 and PSS2 utilize phosphatidylcholine (PC) and PE, respectively, as substrates to synthesize PS [8–12], while in yeast, CHO1 uses CDP-diacylglycerol (CDP-DAG) as the precursor [13, 14]. In mammals, *ex vivo* alteration of the expression of PS metabolic enzymes is the major approach to revealing the functions of PS and the enzymes related to its metabolism. However, disturbing one enzymatic reaction may cause differential changes in the levels of substrate, product and product-derived metabolites. It is hard to tease apart the specific contribution of individual metabolite changes *in vivo*. Moreover, there is strong redundancy and compensation of PS metabolic pathways in mammals. For instance, in mice, deficiency of PSS1 or PSS2 is viable, while double deficiency of PSS1 and PSS2 is embryonic lethal [9, 10]. Therefore, although extensive *ex vivo* studies have revealed many functions of PS at different subcellular locations [15–17], the detailed underlying mechanisms and *in vivo* functions of PS remain to be fully understood.

Besides the metabolic enzymes, the intracellular lipid trafficking routes are also important for the function of phospholipids [18–22]. From the ER, PS is transported to the PM by oxysterol-binding protein (OSBP) family proteins utilizing the phosphatidylinositol 4-phosphate (PI4P) gradient between the ER and PM, which is generated by PI4KIIIα, also known as PI4Kα or Stt4 [23, 24]. PS can also be imported into the mitochondria through ER-mitochondrion connections and the Ups2-Mdm35 complex [25–29]. How the cell coordinates different PS transport routes remains to be investigated.

In this study, we identified the sole *Drosophila* phosphatidylserine synthase, PSS, from an *in vivo* RNAi screen for genes affecting lipid storage. Besides an ectopic lipid storage phenotype, *pss RNAi* caused defects in cell growth and mitochondrial integrity, including mitochondrial protein import. We reveal distinct metabolic causes of these phenotypes and, more importantly, we show that there is a balance between PS transport from the ER to the PM and from the ER to mitochondria.

## Results

### Loss of *CG4825* reduces cell size and causes ectopic lipid storage in *Drosophila* salivary gland

**Fig 1:**
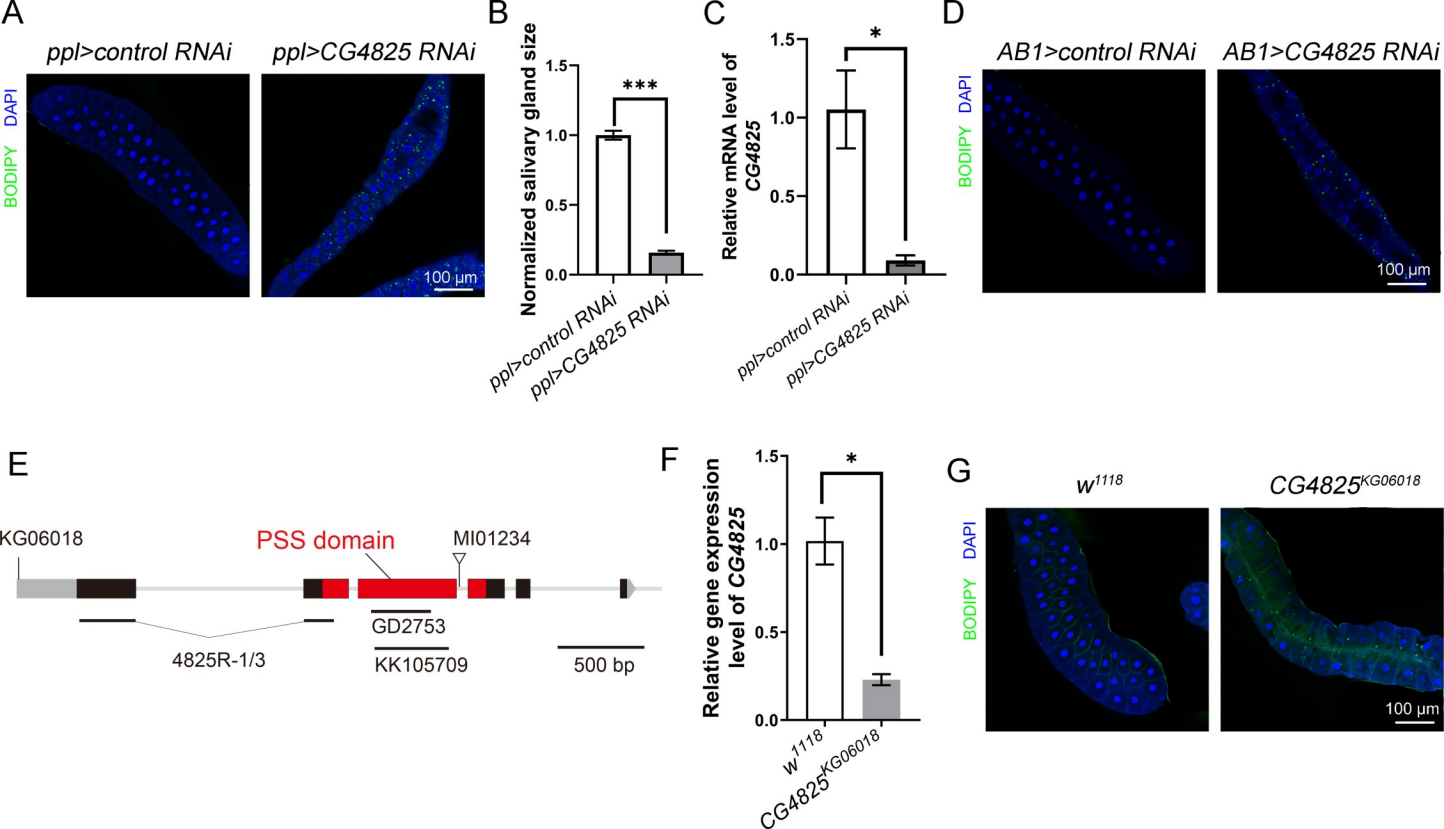
Loss of function of *CG4825* affects lipid storage and cell growth. (A) Knockdown of *CG4825* in salivary glands reduces cell size and causes lipid droplet accumulation in 3^rd^ instar larval salivary glands. (B) Quantification of the salivary gland size (n = 5 for each group). (C) The knockdown efficiency of *ppl>CG4825 RNAi* determined by qRT-PCR (each group has three biological repeats with 25 flies in each repeat). (D) Staining of lipid droplets in 3^rd^ instar larval salivary glands with knockdown of *CG4825* driven by *AB1-GAL4*. (E) The gene structure of *CG4825*. The UTR regions are shown as grey boxed regions while the black and red boxes represent the protein coding region. The red boxed regions indicate the predicted PSS domain. The transposon insertion sites of *CG4825*^*KG06018*^ and *CG4825*^*MI01234*^ and the RNAi target regions are marked. (F) The mRNA level of *CG4825* in wild-type and *CG4825*^*KG06018*^ mutant 3^rd^ instar larval salivary glands (each group has three biological repeats, and each repeat contains 25 flies). (G) Accumulation of lipid droplets in *CG4825*^*KG06018*^ mutant 3^rd^ instar larval salivary gland cells. In A, D and G, BODIPY (green) stains lipid droplets and DAPI (blue) stains the nuclei. In B, C and F, data are shown as mean ± SEM, and were compared with the unpaired Welch Two Sample *t*-test. * p < 0.05, ** p < 0.01, *** p < 0.001. Scale bar represents 100 μm.

We previously performed an RNAi screen in *Drosophila* 3^rd^ instar larval salivary gland and fat body for aberrant lipid storage by using *pumpless-Gal4 (ppl-GAL4)* as a driver to achieve specific RNAi expression in salivary gland and fat body[30]. In the screen, dissected 3^rd^ instar larval salivary glands were stained with the neutral lipid dye BODIPY or Nile red. We found that *ppl-GAL4-*driven RNAi knockdown of *CG4825* (*ppl*>*CG4825*^*KK105709*^ *RNAi*) causes ectopic lipid accumulation in salivary gland and reduces salivary gland size compared with the *ppl-GAL4* control or control RNAi group (*ppl>control RNAi*) (Fig 1A and 1B and S1 Fig). The reduced organ size could be the result of reduced cell number and/or decreased cell size. We found that the salivary gland cell number is not changed in *CG4825 RNAi* (S1 Fig), while the cell size is reduced significantly (S1 Fig). This suggests a defect in cell growth but not cell proliferation. Compared with the control, RNAi dramatically reduces the mRNA level of *CG4825* in 3^rd^ instar larval salivary gland (Fig 1C), confirming the knockdown effect.

Three other independent *ppl-GAL4-*driven *CG4825* RNAi lines (*CG4825*^*GD2753*^, *CG4825*^*NIG4825R-1*^ and *CG4825*^*NIG4825R-3*^) resulted in similar phenotypes, validating the specific effects of *CG4825* knockdown (S1 Fig). Because *ppl-GAL4* is highly expressed in both fat body and salivary gland, we also used the salivary gland-specific driver *AB1-GAL4* to knock down the expression of *CG4825* in the salivary gland but not the fat body. Similar to *ppl-GAL4*-mediated RNAi knockdown, *AB1>CG4825*^*KK105709*^ *RNAi* increases salivary gland lipid storage and reduces cell size (Fig 1D).

*CG4825 RNAi* driven by ubiquitously expressed *tub-GAL4* leads to lethality at the 1^st^ instar larval stage, which indicates that *CG4825* is essential for viability. Besides these RNAi lines, we also examined *CG4825* mutants. *CG4825*^*MI01234*^ is a loss-of-function mutant of *CG4825* (Fig 1E), and the homozygous *CG4825*^*MI01234*^ mutation is lethal during the 1^st^ instar larval stage, which precludes us from directly examining the ectopic lipid phenotype in the salivary gland. *CG4825*^*KG06018*^ is a hypomorphic allele of *CG4825* with a transposon element inserted into the transcription start site (Fig 1E). The *CG4825* transcription level in *CG4825*^*KG06018*^ salivary gland is reduced to about 20% of that of wild type (Fig 1F). Importantly, similar to C*G4825 RNAi*, there are ectopic lipid droplets in *CG4825*^*KG06018*^ 3^rd^ instar larval salivary gland cells (Fig 1G). Together, these results demonstrate the tissue-autonomous function of *CG4825* in regulating cell growth and lipid storage.

### *CG4825* encodes the sole *Drosophila* phosphatidylserine synthase (PSS)

**Fig 2:**
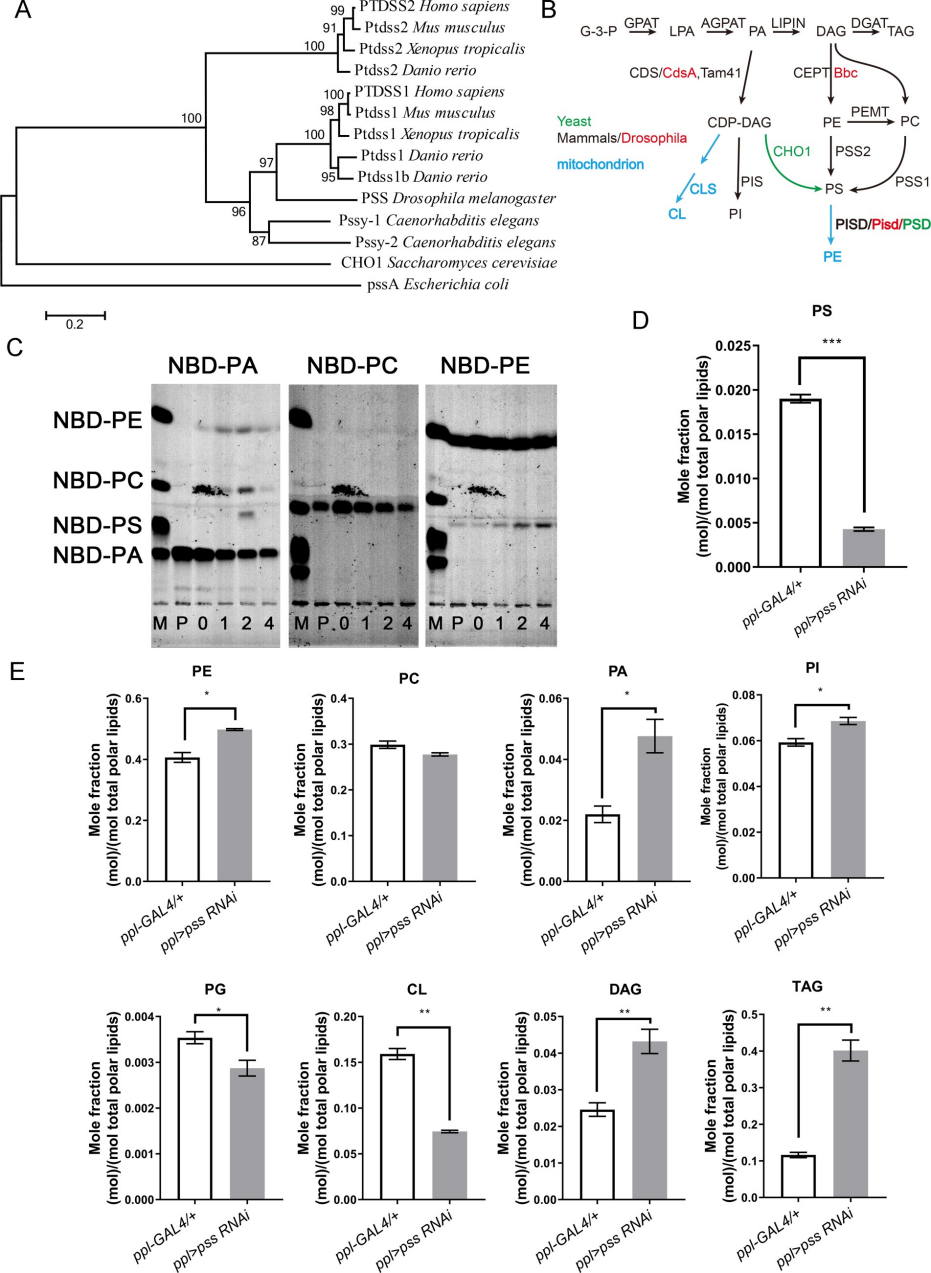
CG4825 is the sole *Drosophila* phosphatidylserine synthase (PSS). (A) The evolutionary phylogenetic tree of PSS and its homologs in different species. The scale bar indicates amino acid divergence between sequences. Bootstrap number is 1,000 for resampling. (B) Schematic of the phospholipid and glycerolipid synthesis pathways. The black arrows indicate the common pathways in different species, the green arrow shows the pathway in yeast, the red arrows indicate the pathways in mammals, and the blue arrows indicate the pathways that occur in mitochondria. (C) S2 cells were cultured with medium containing 10 μM NBD-PA, NBD-PC, or NBD-PE for pulse-chase analysis. The S2 cells were harvested before the pulse-chase treatment (P) and 0, 1, 2, and 4 hours after pulse-chase. The extracted total lipids from S2 cells were separated on TLC plates for 1~2 hour. NBD-PE is converted to NBD-PS while NBD-PC is not. M, marker. Noted that a contaminating signal, which is likely from the plate of Typhoon 9500 imager, is present in all experiments. (D-E) Lipid profiling of control and *pss RNAi* 3^rd^ instar larval salivary glands. The levels of the lipid species were normalized by calculating the mole fraction of the total polar lipids. In *pss RNAi*, the levels of PS, PG and CL are reduced, the levels of PE, PA, PI, DAG and TAG are increased, and the level of PC is unchanged. Data are shown as mean ± SEM. Data were compared with the unpaired Welch Two Sample *t*-test. * p < 0.05, ** p < 0.01, *** p < 0.001.

CG4825 contains a phosphatidylserine synthase (PSS) domain and is conserved from yeast to mammals based on protein sequence alignment (Fig 1E and Fig 2A). From bacteria to mammals, there are different PSS proteins for the synthesis of PS from different substrates (Fig 2B). CG4825 is the only PSS domain-containing protein in *Drosophila*, so we refer to it as PSS hereafter. *Drosophila* PSS is similar to mammalian PSS1 in the phylogenetic tree (Fig 2A). It has been reported that PSS activity is required for embryonic viability in mice [9, 10]. This is consistent with the lethal phenotype in *pss*^*MI01234*^ mutants and the global knockdown of *pss*, and suggests that *Drosophila* PS synthesis is mainly through *pss*.

To determine which substrate is used by *Drosophila* PSS, we pulse-traced NBD-labeled PA (NBD-PA), PC (NBD-PC) and PE (NBD-PE) in *Drosophila* S2 cells and used thin layer chromatography (TLC) to examine their conversion. The NBD-labeled PA was converted to PE, PC and PS (Fig 2C). Labeled PS appeared later than PE and PC, which suggests that it may be derived from PE or PC. Treating cells with NBD-PC did not yield any labeled PS (Fig 2C), which indicates that S2 cells may not be able to convert PC to PS. However, NBD-PS did appear after the cells were pulse-labeled with NBD-PE (Fig 2C). Together, these results suggest that PA can be converted to PE and subsequently to PS in *Drosophila* S2 cells.

We further analyzed whether RNAi of *pss* affects the level of PS *in vivo*. We dissected wild-type and *pss RNAi* salivary glands and measured the levels of PS and other lipids through lipidomic profiling. As expected, the level of PS is dramatically reduced in *pss RNAi* to around 20% of wild type (Fig 2D). The level of PE is increased, while the PC level is not significantly changed (Fig 2E). Moreover, along with the marked reduction of PS, the levels of PA and PI are significantly increased, while the levels of phosphatidylglycerol (PG) and cardiolipin (CL) are reduced (Fig 2E). The lipidomic data also show that the levels of triacylglycerol (TAG) and diacylglycerol (DAG) are increased in *pss RNAi*, consistent with the BODIPY staining result (Fig 2E). Put together, these results indicate that as the sole PS synthase in *Drosophila*, PSS, likely uses PE as the substrate for PS synthesis.

### *pss* knockdown depletes plasma membrane Akt and reduces cell growth, at least in part, via the insulin pathway

**Fig 3:**
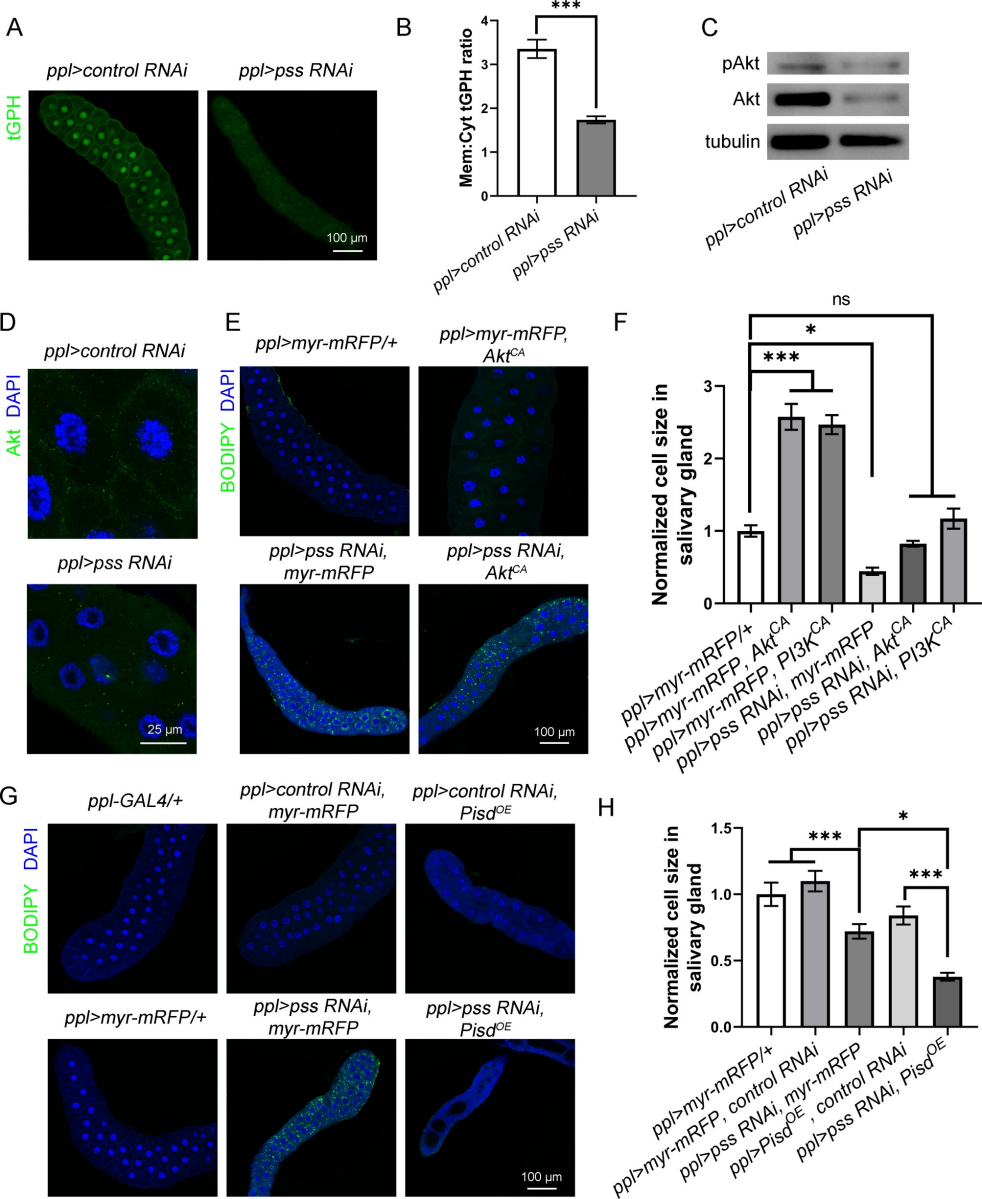
The cell growth defect of *pss RNAi* is caused by impaired insulin pathway activity. (A) Images of the tGPH reporter in control and *pss RNAi* 3^rd^ instar larval salivary glands. The same laser power and exposure time were used during the imaging. Scale bar represents 100 μm. (B) Quantification of the PM:cytoplasm PIP_3_ ratio in control and *pss RNAi* salivary glands (n = 5). Data are shown as mean ± SEM and were compared with Welch Two Sample *t*-test. *** p < 0.001. (C) Western blot of Akt and pAkt in *ppl>control RNAi*, and *pss RNAi* 3^rd^ instar larval salivary glands. Three replicates were performed and a representative result from one replicate is shown here. A total of 10 μg protein was loaded. (D) Immunofluorescent staining of Akt in 3^rd^ instar larval salivary gland cells. Scale bar represents 25 μm. (E) Overexpression of Akt^CA^ rescues the reduced salivary gland size, but not the ectopic lipid accumulation, of *pss RNAi*. Scale bar represents 100 μm. (F) Quantification of the size of salivary gland cells with RNAi of *pss* and over-expression of Akt (n = 5 for each group). Data are shown as mean ± SEM. Data were compared with One-way ANOVA. Multiple comparisons of means were conducted with Tukey Contrasts. * p < 0.05, *** p < 0.001, ns: not statistically significant. (G) Overexpression (OE) of *Pisd* (*CG5991*) in *pss RNAi* suppresses the lipid accumulation and enhances the cell size reduction. In E and F, BODIPY (green) stains lipid droplets and DAPI (blue) stains the nuclei. Scale bar represents 100 μm. (H) Quantification of the size of salivary gland cells in different genetic backgrounds (n = 5 for each group). Data are shown as mean ± SEM. Data were compared with One-way ANOVA. Multiple comparisons of means were conducted with Tukey Contrasts. * p < 0.05, *** p < 0.001.

We next explored the mechanisms underlying *pss*-mediated cell growth and neutral lipid homeostasis. Previous reports show that the insulin pathway regulates *Drosophila* salivary gland cell growth [30, 31]. We examined the activity of the insulin pathway. tGPH is a GFP reporter that reflects insulin pathway activity through the PM:cytoplasm ratio of GFP signal [31]. We found that the plasma membrane tGPH signal is decreased and the PM:cytoplasm ratio of tGPH signal is reduced in the 3^rd^ instar larval salivary gland of *pss RNAi* compared to controls (Fig 3A and 3B). The levels of Akt, a key component in the insulin pathway, and phosphorylated Akt (pAkt) also reflect insulin pathway activity, so we examined them by western blotting. The levels of both Akt and pAkt are significantly reduced in the 3^rd^ instar larval salivary gland in *pss RNAi* (Fig 3C). Compared to the dramatic reduction of protein level, *pss RNAi* only slightly reduces the transcription of Akt in salivary glands (S2 Fig), suggesting a likely effect of *pss* RNAi on Akt translation or stability.

The membrane recruitment of Akt by binding to PIP_3_ in the PM is the key to insulin pathway activation. In addition, a recent study showed that Akt binds to both PIP_3_ and PS in the PM for its full activation [32]. Since the level of PS is greatly reduced in *pss RNAi*, we hypothesized that with the reduction of plasma membrane PS, *pss RNAi* may impair Akt membrane recruitment. We tested this by immunostaining Akt. The Akt signals exhibit punctate patterns in both the cytosol and cell periphery, presumably the PM, in control cells. Although the cytosolic Akt puncta are still present in *pss RNAi* cells, interestingly, the Akt signals in the PM are lost in *pss RNAi* cells, indicating a decreased membrane recruitment of Akt in *pss RNAi* (Fig 3D).

We also tested the genetic interactions of *pss* and insulin pathway components. If impaired insulin pathway activity contributes to the small salivary gland cells in *pss RNAi*, elevating insulin pathway activity should suppress the *pss RNAi* cell growth defect. Indeed, the small cell phenotype in *pss RNAi* is suppressed by overexpressing either constitutively active Akt (Akt^CA^) or a constitutively active PI3K (PI3K^CA^), which is a PIP_2_ kinase acting upstream of Akt (Fig 3E, 3F and S2B). Notably, the overgrowth phenotype of Akt^CA^ or PI3K^CA^ overexpression alone are also suppressed by *pss* RNAi. Since neither RNAi nor overexpression represents a true genetic null, the additive effect of the doubles can still be explained by *pss* and *Akt* affecting the same genetic pathway. Alternatively, it is possible that other defects in *pss RNAi* may also affect cell growth. Nevertheless, considering the additive effect of the genetic doubles and the reduced membrane Akt signal in *pss RNAi*, these results indicate that *pss RNAi* depletes plasma membrane Akt and reduces cell growth, at least in part, via the insulin pathway.

### Overexpressing phosphatidylserine decarboxylase enhances the cell growth defect and suppresses the ectopic lipid storage phenotype of *pss* knockdown

If a reduced level of plasma membrane PS is the cause of impaired insulin pathway activity in *pss RNAi*, elevating the level of plasma membrane PS might rescue the small cell size phenotype, and further reduction of the PS level should exacerbate the reduced cell size phenotype in salivary glands. Phosphatidylserine decarboxylase, which is encoded by the *Pisd* gene in mice and by *PSD* in yeast (Fig 2B), is localized in mitochondria under normal condition and converts PS to PE. We examined the phenotype of *pss RNAi* with knockdown or overexpression of *Drosophila Pisd* (*CG5991*). We hypothesized that in *pss RNAi* salivary glands, RNAi of *Pisd* may increase the PS level in the ER and subsequently restore the plasma membrane PS level, while overexpression of *Pisd* may further decrease the plasma membrane PS level. We found that RNAi of *Pisd* did not suppress the cell size phenotype caused by *pss RNAi* (S2 Fig), although the RNAi reduced the expression level of *Pisd* (S2 Fig). However, overexpressing *Pisd* further reduced the size of 3^rd^ instar larval salivary gland cells in *pss RNAi* (Fig 3G and 3H). Together, these results provide additional evidence that a reduced level of plasma membrane PS in *pss RNAi* impairs insulin pathway activity and affects cell growth.

Is the ectopic lipid storage phenotype in *pss RNAi* also caused by reduced PS and impaired insulin pathway activity? Interestingly, *Pisd* overexpression completely suppresses the ectopic lipid accumulation phenotype of *pss RNAi* (Fig 3G), which demonstrates that these two phenotypes are caused by different mechanisms. Accordingly, although elevating insulin pathway activity by overexpression of either Akt^CA^ or PI3K^CA^ suppresses the cell growth defect, it only marginally reduces the ectopic lipid storage in *pss RNAi* (Figs 3E and S2B).

### Loss of *pss* affects mitochondrial protein import and mitochondrial integrity

The above results prompted us to further investigate the underlying mechanism of *pss RNAi*-induced ectopic lipid accumulation. Another important trafficking route of PS, besides moving from the ER to the PM, is its import from the ER into mitochondria. Previous reports showed that mitochondrial PS is mainly used for the production of mitochondrial PE, which is important for mitochondrial morphology and function [26, 33]. In the lipidomic analysis of *pss RNAi*, our attention was also drawn to mitochondria by the marked reduction of CL (Fig 2E), a special phospholipid that is highly enriched in mitochondria. Mitochondrial fatty acid oxidation is important for the catabolism of lipids and therefore disturbing the function of mitochondria may lead to lipid accumulation[34].

**Fig 4:**
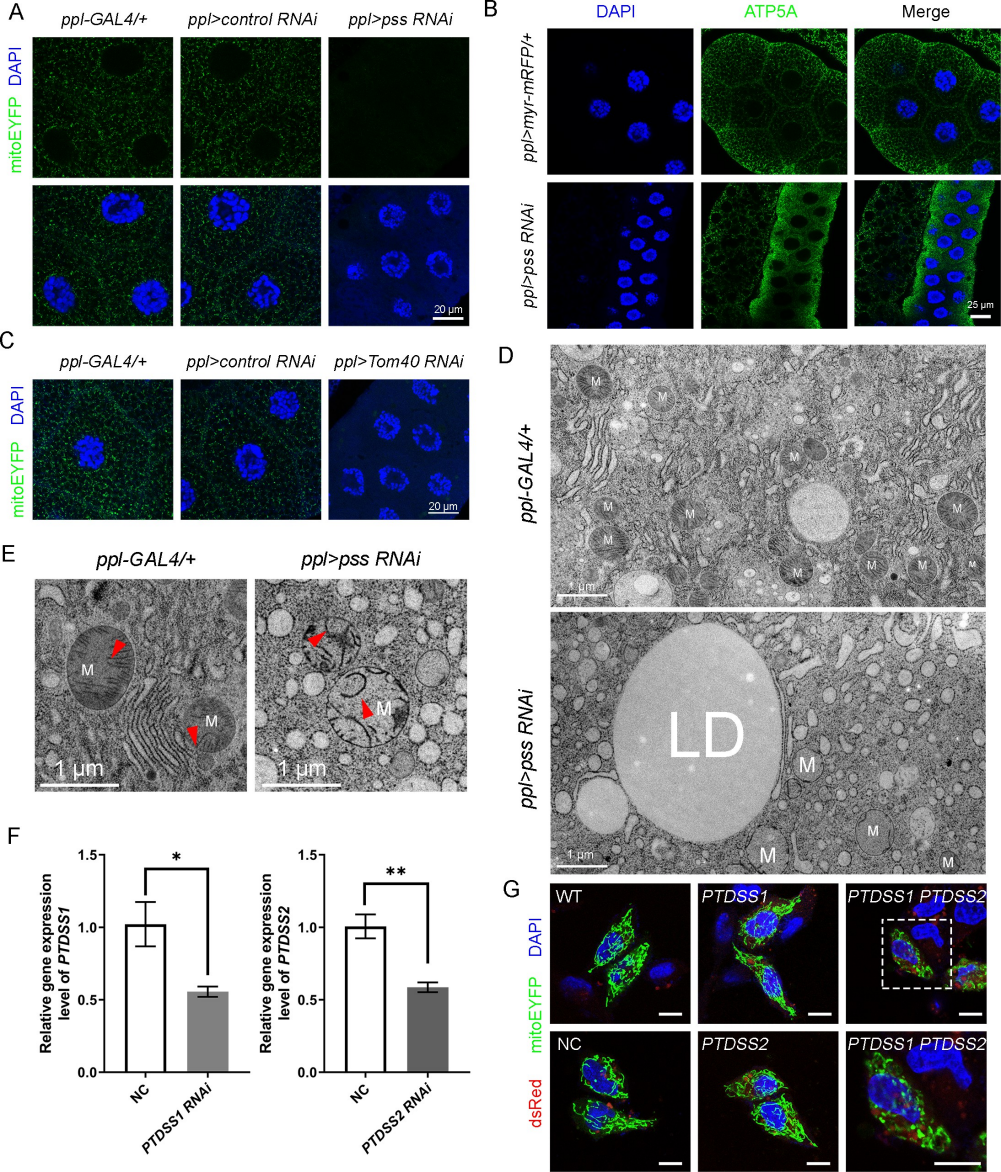
*pss RNAi* affects mitochondrial integrity. (A) Images of mitoEYFP in 3^rd^ instar larval salivary glands of *ppl-Gal4/+*, *ppl>control RNAi* and *pss RNAi*. Green (mitoEYFP), mitochondria; blue (DAPI), nuclei. Scale bar represents 20 μm. (B) Immunofluorescent staining of mitochondria with anti-ATP5A antibody in 3^rd^ instar larval salivary glands. The nuclei are labeled with DAPI. Scale bar represents 25 μm. (C) Fluorescence signal of mitoEYFP reporter in 3^rd^ instar larval salivary glands of *ppl-GAL4/+*, *ppl>control RNAi* or *Tom40 RNAi*. The mitoEYFP signal is absent in *Tom40 RNAi* salivary gland cells. Scale bar represents 20 μm. (D-E) EM images of mitochondria in 3^rd^ instar larval salivary glands of *ppl-GAL4/+* and *pss RNAi*. The red arrows mark cristae. Scale bar represents 1 μm. M, mitochondria; LD, lipid droplet. (F) The knockdown efficiency of human *PTDSS1* and *PTDSS2* in HeLa cells (n = 3). Data are shown as mean ± SEM. Data were compared with the unpaired Welch Two Sample *t*-test. * p < 0.05, ** p < 0.01. (G) Images of mitoEYFP in HeLa cells with RNAi of human *PTDSS1* and *PTDSS2*. The tubular structures are fragmented in cells with double RNAi of human *PTDSS1* and *PTDSS2*. Scale bar represents 10 μm.

Does knockdown of *Drosophila pss* affect mitochondria? We used a mitoEYFP reporter to label the mitochondria *in vivo* [35]. In control 3^rd^ instar larval salivary glands, mitoEYFP appears as fluorescent puncta (Fig 4A). Surprisingly, the fluorescent signal is almost completely absent in *pss RNAi* (Fig 4A). MitoTimer, a GFP-based mitochondrial marker [36], shows the same phenotype in *pss RNAi* (S3 Fig). We found that *pss RNAi* does not significantly affect the transcription of mitoEYFP in salivary glands (S3 Fig). In addition, *pss RNAi* does not prevent the expression of other GFP or myr-mRFP reporters tested (S3 Fig). To explore whether the lack of the mitoEYFP fluorescent signal is due to the absence of mitochondria in *pss RNAi*, we immunostained mitochondria with an antibody against ATP5A, the alpha subunit of mitochondrial ATP synthase. The ATP5A signal is present in both control and *pss RNAi* salivary gland cells (Fig 4B), indicating that mitochondria are present in *pss RNAi* cells. The ATP5A signal appears as punctate structures in wild type, while in *pss RNAi*, the signal is more condensed, which could be due to packed mitochondria in small cells (Fig 4B). Both mitoEYFP and MitoTimer utilize the mitochondrial targeting signal of human COX8A. Together, these results raise the possibility that mitochondria in *pss RNAi* cells have a defect in the import of certain mitochondrial proteins and that mitochondrially targeted fluorescent proteins are likely degraded if they are not properly imported. To test this, we performed RNAi of Tom40, a mitochondrial outer membrane protein which is known to function in mitochondrial protein import [37], and examined the mitoEYFP pattern. Indeed, similar to *pss RNAi*, the fluorescent signal of mitoEYFP is gone in *Tom40 RNAi* (Fig 4C), which is consistent with a previous report [38].

To achieve a better understanding of mitochondrial integrity in *pss RNAi*, we performed ultra-structural analysis of 3^rd^ instar larval salivary gland by electron microscopy (EM). In control cells, we observed round mitochondria with clear smooth cristae (Fig 4D). Within mitochondria, the cristae were often oriented in parallel and the matrix was uniformly stained (Fig 4E). In *pss RNAi* cells, consistent with the anti-ATP5A antibody staining result, many mitochondria can be found (Fig 4D). However, the mitochondrial morphology was grossly aberrant compared to control. The cristae were disorganized and were often bent, looped and branched (Fig 4E). The crista junction width is increased and the electron density is higher at the junction sites (S3 Fig). The *pss RNAi* cells also contained large lipid droplets, consistent with the BODIPY staining results (Fig 4D). Together, these results indicate that with reduced PS import into mitochondria, loss of *pss* affects mitochondrial protein import and mitochondrial integrity.

We also examined the mitochondrial phenotype caused by knocking down the mammalian PS synthases, *PTDSS1* and *PTDSS2*, in HeLa cells. The mRNA levels of human *PTDSS1* and *PTDSS2* are significantly reduced by RNAi (Fig 4F). When we labeled the mitochondria with mitoEYFP, we found that the mitoEYFP signal *per se* is not affected by all the RNAi treatments. The tubular mitochondria labeled by mitoEYFP are not obviously affected by *PTDSS1* or *PTDSS2* single RNAi (Fig 4G). However, the mitochondria are fragmented in cells with double knockdown of *PTDSS1* and *PTDSS2* (Fig 4G). This indicates that loss of PS synthesis in HeLa cells affects mitochondrial morphology, consistent with previous reports [33].

### The ectopic lipid storage phenotype in *pss RNAi* is likely due to the metabolic shift from phospholipid synthesis to neutral lipid synthesis

**Fig 5:**
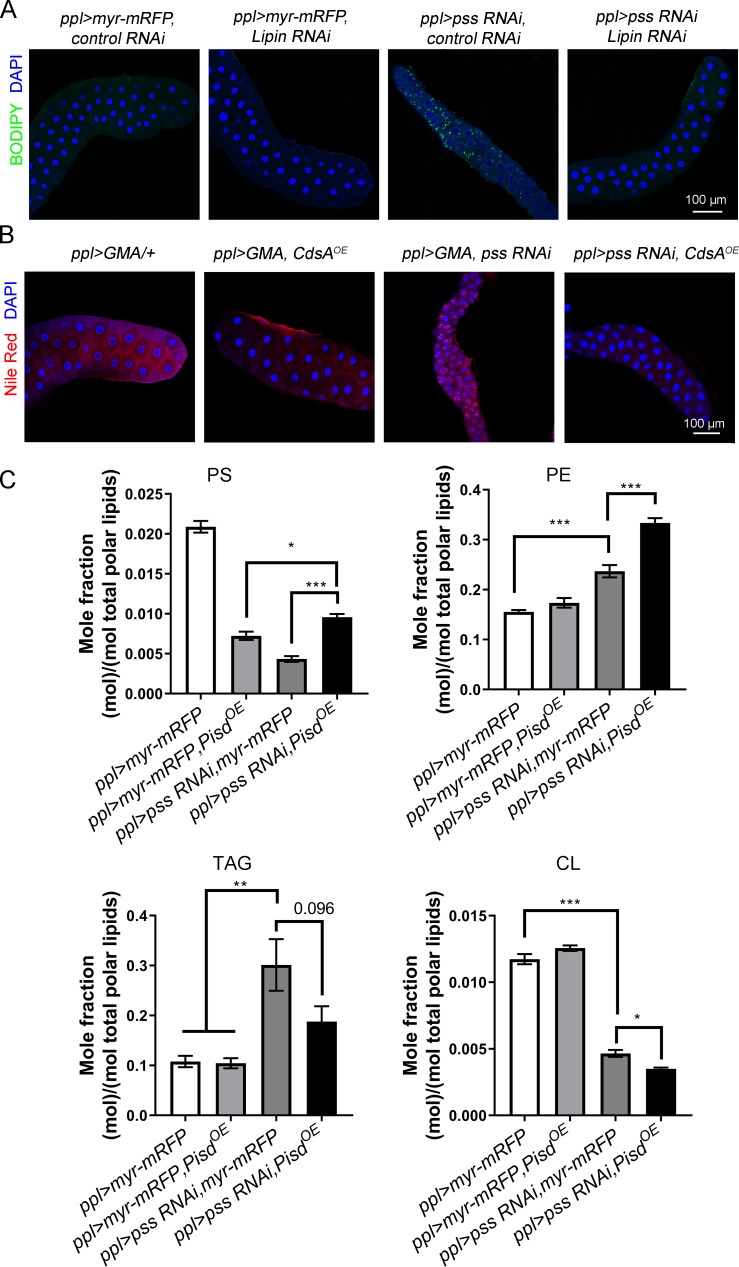
The metabolic shift from phospholipid synthesis to TAG synthesis contributes to ectopic lipid storage in salivary glands with *pss RNAi*. (A) Staining of lipid droplets with BODIPY in 3^rd^ instar larval salivary gland with RNAi of *Lipin*. DAPI stains nuclei. Scale bar represents 100 μm. (B) Staining of lipid droplets with Nile Red shows that overexpression (OE) of *CdsA* suppresses the lipid accumulation in 3^rd^ instar larval salivary glands of *pss RNAi*. DAPI stains nuclei. Scale bar represents 100 μm. (C) Lipid profiling of 3^rd^ instar larval salivary glands of control, *pss RNAi*, *Pisd*^*OE*^, and *pss RNAi* with *Pisd*^*OE*^. Data are shown as mean ± SEM. Data were compared with one-way ANOVA. Multiple comparisons of means were conducted with Tukey Contrasts. * p < 0.05, ** p < 0.01, *** p < 0.001.

What is the relationship between mitochondrial abnormality and ectopic lipid storage? The increased levels of PE, DAG and PA, along with the reduced conversion of PE to PS (Fig 2E), may provide an explanation for the ectopic lipid storage in *pss RNAi*. In our previous study [39], a similar ectopic lipid storage phenotype was found in *CdsA RNAi* and *bbc RNAi*, which shifted PA-(CDP-DAG) synthesis to PA-DAG synthesis and DAG-PE synthesis to DAG-TAG synthesis, respectively. Therefore, it is possible that the ectopic lipid storage phenotype in *pss RNAi* is caused by the metabolic shift from phospholipid synthesis, namely DAG-PE-PS, to neutral lipid synthesis (Fig 2B). To test this hypothesis, we performed genetic analysis. RNAi of *Lipin*, the gene encoding phosphatidic acid phosphatase which generates DAG from PA, fully suppresses the lipid storage phenotype of *pss RNAi* (Fig 5A). Similarly, *CdsA* overexpression completely suppresses the ectopic lipid phenotype of *pss RNAi* (Fig 5B). Together, these results indicate that the metabolic shift from phospholipid synthesis to neutral lipid synthesis in *pss RNAi* likely results in ectopic lipid storage.

We also analyzed the lipid profile in *pss RNAi* with *Pisd* overexpression. The level of TAG is marginally reduced, although not statistically significant, in cells with *pss RNAi* and *Pisd* overexpression (Fig 5C), which is consistent with our observation that lipid droplets are not found in cells of *pss RNAi* with *Pisd* overexpression. *Pisd* overexpression further increases the level of PE in *pss RNAi* salivary gland, indicating that *Pisd* overexpression increases overall lipid flow from PS to PE (Fig 5C). Intriguingly, the PS level in salivary gland cells with RNAi of *pss* and overexpression of *Pisd* is marginally higher compared to *Pisd* overexpression alone or *pss* single RNAi (Fig 5C). The lipidomic data also show a further reduction of the CL level in the *pss RNAi* salivary gland with overexpression of *Pisd* (Fig 5C).

### Decreasing lipid storage or promoting cell growth cannot rescue the mitochondrial dysfunction caused by *pss RNAi*

**Fig 6:**
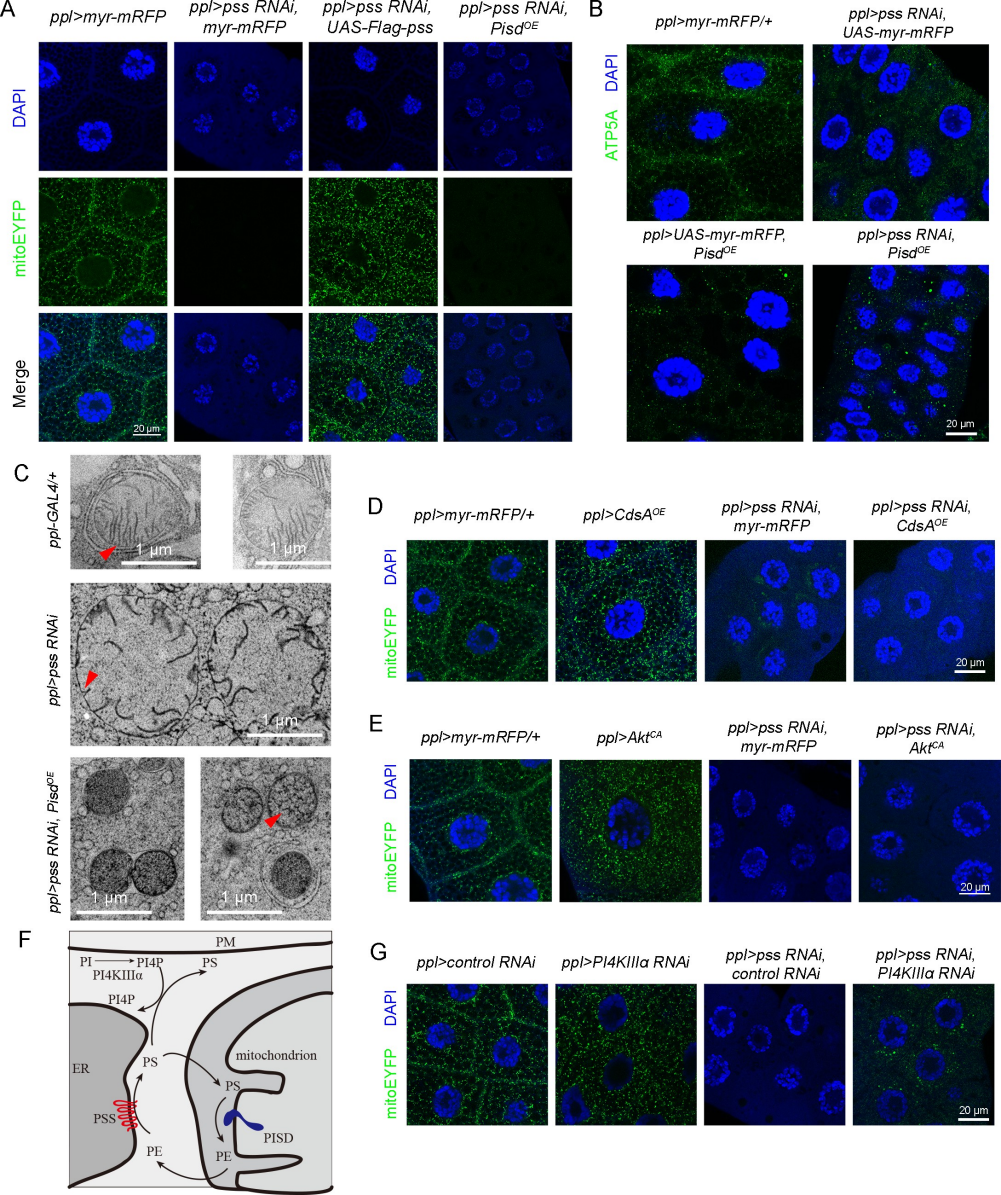
The mitochondrial defect of *pss RNAi* is not rescued by *Pisd* overexpression. (A) Fluorescence images of mitoEYFP in 3^rd^ instar larval salivary glands of different genetic backgrounds. The absence of mitoEYFP signal in *pss RNAi* salivary glands is not rescued by *Pisd* (*CG5991)* overexpression. mitoEYFP (green) marks mitochondria and DAPI (blue) stains the nuclei. Scale bar represents 20 μm. (B) Immunostaining of mitochondria with anti-ATP5A antibody in 3^rd^ instar larval salivary glands of different genetic backgrounds. Anti-ATP5A antibody (green) marks mitochondria and DAPI (blue) stains the nuclei. Scale bar represents 20 μm. (C) The EM structure of mitochondria in 3^rd^ instar larval salivary gland cells of control, *pss RNAi*, and *pss RNAi* with *Pisd* overexpression (*Pisd*^*OE*^). Scale bar represents 1 μm. Red arrows in *ppl-GAL4/+* and *ppl>pss RNAi* salivary gland cells mark the crista junction sites, and the red arrow in the salivary gland cell with *pss RNAi* and *Pisd*^*OE*^ marks the filamentous meshes or crista fragments. (D) Images of mitoEYFP in 3^rd^ instar larval salivary glands of different genetic backgrounds. Overexpression of *CdsA* does not restore the mitoEYFP signal in salivary glands of *pss RNAi*. Scale bar represents 20 μm. (E) Images of mitoEYFP in 3^rd^ instar larval salivary glands of different genetic backgrounds. The loss of mitoEYFP signal in *pss RNAi* is not rescued by the overexpression of Akt^CA^. Scale bar represents 20 μm. (F) Illustration of the cellular PS transport pathways. (G) Images of mitoEYFP in 3^rd^ instar larval salivary glands of different genetic backgrounds. The loss of mitoEYFP signal in *pss RNAi* is partially rescued by *PI4KIIIα RNAi*.

Since *Pisd* overexpression completely suppresses the ectopic lipid accumulation phenotype of *pss RNAi*, we next examined whether *Pisd* overexpression rescues the mitochondrial defect of *pss RNAi*. Overexpression of *Pisd* did not restore the mitoEYFP signal in *pss RNAi* (Fig 6A). In addition, compared to wild type or *pss RNAi* alone, some large ATP5A-positive puncta appeared in *pss RNAi* with *Pisd* overexpression (Fig 6B). We also conducted ultra-structural analysis by EM (Fig 6C). Compared with *pss RNAi*, the structural abnormalities of mitochondria seem much worse in *pss RNAi* with *Pisd* overexpression. The regular tubular crista structures have almost completely disappeared. Instead, filamentous mesh resembling matrix condensation or crista fragments were found in the mitochondrial matrix (Fig 6C). In addition, autophagsome was frequently found (Fig 6C). These results indicate that although *Pisd* overexpression rescued the ectopic lipid storage phenotype of *pss RNAi*, it did not rescue the mitochondrial defects. Similarly, overexpression of *CdsA* does not rescue the mitoEYFP import defect in *pss RNAi*, even though it completely rescues the ectopic lipid storage phenotype (Fig 6D). Along the same line, we found that overexpression of *Akt*^*CA*^ does not rescue the loss of the mitoEYFP signal (Fig 6E), but it does rescue the cell growth defect of *pss RNAi* (Fig 3E). Together, these results indicate that PSS regulates mitochondrial function and cell growth or lipid storage through distinct mechanisms.

### Reducing PS transport from the ER to PM by loss of PI4KIIIα partially rescues the mitochondrial defects of *pss RNAi*

None of the above manipulations (overexpression of *CdsA*, *Akt*^*CA*^ or *Pisd*) rescues the mitochondrial defects of *pss RNAi*. This observation is consistent with the idea that reduced PS import into mitochondria is probably the underlying cause of the mitochondrial defects, because overexpression of *CdsA*, *Akt*^*CA*^ or *Pisd* is unlikely to increase the level of mitochondrial PS. After its synthesis in the ER, PS is transported to other cellular membranes such as PM, mitochondria and Golgi. It is unknown whether these different PS transport routes are coordinated. In particular, it is not known whether there is a balance between different PS transport routes and, if there is, whether disrupting one PS transport route may promote other PS transport routes. If that is the case, blocking PS transport from the ER to the PM in *pss RNAi* may divert more PS to mitochondria and therefore suppress the mitochondrial defect of *pss RNAi*. PS transport from the ER to the PM relies on the PI4P gradient between the PM and ER [5, 6]. At the PM, PI4KIIIα phosphorylates PI to PI4P (Fig 6F). RNAi of *PI4KIIIα* may reduce the level of PI4P in the PM, leading to loss of the PM-ER PI4P gradient required for PS transport from the ER to PM. We knocked down the expression of *PI4KIIIα* in *pss RNAi* salivary gland cells. Interestingly, the loss of the mitoEYFP signals in *pss RNAi* salivary gland cells is partially reversed by RNAi of *PI4KIIIα*, suggesting that reducing PS transport from the ER to PM may induce a compensatory increase in PS transport from the ER to mitochondria and rescue the mitochondrial defects of *pss RNAi* (Fig 6G). Furthermore, this result indicates that there is a balance between PS transport from the ER to PM and from the ER to mitochondria.

## Discussion

There are numerous difficulties in revealing the *in vivo* cellular and physiological roles of phospholipids, including redundancy of metabolic genes, interconnected metabolic pathways, and different contributions of substrate, product and further metabolites derived from the product. In this study, through genetics and lipidomic analysis, we reveal that distinct mechanisms underlie the pleiotropic cellular defects caused by knocking down *Drosophila* PS synthase, PSS. Our detailed phenotypic and mechanistic analyses of *pss* knockdown provide a clear example of how altering lipid homeostasis contributes to different cellular phenotypes.

**Fig 7:**
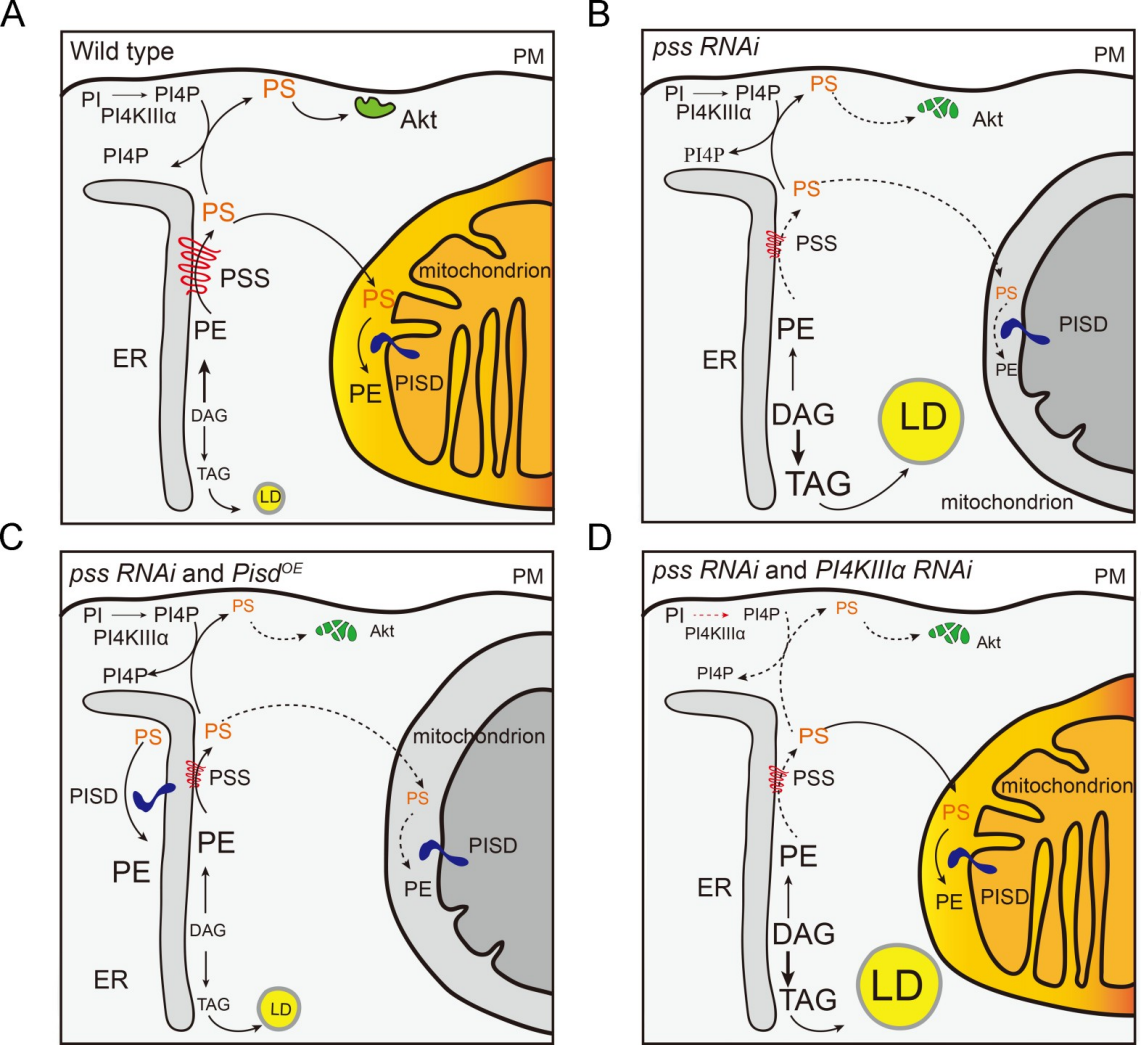
Proposed models of the mechanisms underlying the different *pss RNAi* phenotypes. (A-B) Loss of *pss* function leads to reduced Akt, increased DAG and TAG, and impaired mitochondrial structure and function. (C) The overexpression of *Pisd* in *pss RNAi* may lead to increased engagement of phospholipids in the PS-PE-PS cycle locally, probably at the ER or mitochondria. Therefore, *Pisd* overexpression reduces the level of PS at the PM and the amount of DAG available for TAG synthesis, leading to smaller cells and decreased lipid storage compared to *pss RNAi* alone (B). (D) *RNAi* of *PI4KIIIα* rescues the defective mitochondrial import of mitoEYFP in *pss RNAi* by increasing the transport of PS to the mitochondria.

PS can be synthesized from PC or PE in mammals. In *Drosophila*, we did not detect the conversion of NBD-PC to NBD-PS in S2 cells and it is likely that *Drosophila* PSS utilizes PE, the major phospholipid in *Drosophila*, as the substrate for PS synthesis based on the lipidomic results from 3^rd^ instar larval salivary glands (Fig 2D and 2E). We found that *pss* knockdown leads to cell growth defects, ectopic lipid accumulation and loss of mitochondrial integrity. We further showed that these three defects are likely due to different metabolic impacts of *pss* knockdown (Fig 7A–7D). We propose that PSS regulates cell growth, at least in part, via the insulin pathway, by affecting the level of plasma membrane PS and subsequently the membrane recruitment of Akt (Fig 7B). It is also possible that similar to PI4KIII*α* mutants [40], *pss RNAi* leads to a defect in PM integrity which may cause the reduced Akt recruitment in PM. This notion is further supported by the aberrant myr-mRFP localization in the *ppl>pss RNAi* cells (S3 Fig). Interestingly, a previous study in *Drosophila* also found that both Lipin and GPAT are important for insulin pathway activity [41]. Both GPAT and Lipin act upstream of PSS in PS synthesis (Fig 2B). Therefore, it is possible that the level of PS contributes to the GPAT/Lipin-mediated regulation of insulin pathway activity. Considering the additive effect of *pss RNAi* and elevating insulin pathway activity on cell growth, we cannot rule out the possibility that other cellular defects, such as the mitochondrial dysfunction, may also contribute to the abnormal cell growth in *pss RNAi*.

The ectopic lipid storage phenotype of *pss* knockdown is mainly attributed to the metabolic shift of the glycerolipid synthetic program. Glycerophospholipids and TAG share similar synthetic pathways (Fig 2B). Phospholipid homeostasis and lipid storage are highly interconnected [39, 42]. Besides the overall compensatory DAG-TAG metabolic shift, changes in the levels of specific phospholipids, such as PE, PA and PI, may also contribute to ectopic lipid storage in *pss RNAi*. For example, PE acts as a feedback regulator of SREBP-mediated lipogenesis [43]. The increased level of PA may increase the size of lipid droplets, where TAG is stored [44].

The loss of mitochondrial integrity is the most dramatic consequence of *pss* knockdown. Mitochondria exchange lipids with the ER and other organelles. PS is imported into mitochondria for the synthesis of mitochondrial PE, which is known to be important for mitochondrial morphology and function. In flies, *pss RNAi* affects the mitochondrial import of mitoEYFP and the morphology of mitochondria. In mammalian cells, although the mitochondrial import of mitoEYFP is not affected by double knockdown of *PTDSS1* and *PTDSS2*, the morphology of mitochondria is aberrant. A recent study reported that the reduction of mitochondrial PE promotes the proteolysis of mitochondrial proteins and this may explain the aberrant mitochondria in *pss RNAi* salivary gland cells [45]. Previous reports showed that mitochondrial import of preproteins is impaired in PE-depleted [46] or CL-deficient mitochondria [47, 48]. The difference in mitoEYFP mitochondrial import between *pss RNAi* in *Drosophila* and *PTDSS1/PTDSS2* knockdown in mammalian cells may reflect a difference in mitochondrial protein import between flies and mammals or insufficient knockdown of *PTDSS1/PTDSS2* in mammalian cells.

At first glance, it is puzzling that overexpression of *Pisd* rescues the ectopic lipid accumulation phenotype of *pss RNAi*, but not the mitochondrial defects. The lipid metabolic changes in *pss* RNAi with *Pisd* overexpression is intriguing. *Pisd* overexpression should increase the level of mitochondrial PE. Salivary glands are small, and therefore we were unable to isolate mitochondria from them to measure the mitochondrial PE level. However, the total PE level is indeed increased in *pss RNAi* with *Pisd* overexpression. If PS in the mitochondria is used for the synthesis of mitochondrial PE, why then did overexpression of *Pisd* fail to rescue the mitochondrial defect of *pss RNAi*? It is possible that there are unidentified functions of mitochondrial PS in maintaining mitochondrial integrity and protein import. Alternatively, it is possible that the impairment of mitochondrial protein import in *pss RNAi* prevents a sufficient amount of Pisd entering into mitochondria, and Pisd instead stays in the ER (or other locations), leading to increased PE in the ER (Fig 7C). In support of this hypothesis, a recent report showed that PSD (yeast Pisd) localizes and functions in both mitochondria and ER [49]. However, the elevation of PE in the ER seems contradictory to the metabolic shift in *pss RNAi* and the suppression of the ectopic lipid accumulation phenotype in *pss RNAi* by *Pisd* overexpression (Fig 7B and 7C). The slightly increased level of PS in *pss RNAi* with *Pisd* overexpression compared to *pss RNAi* alone further complicates the analysis. It appears that the elevated PE level caused by *Pisd* overexpression also increases the flow from PE to PS, resulting in the slightly increased PS level in *pss RNAi* with *Pisd* overexpression. It is possible that when *Pisd* is overexpressed, more phospholipids are engaged in the PS-PE-PS cycle locally, probably at the ER or mitochondria. To achieve a full understanding of the underlying metabolic flow changes, organelle-specific lipid profiling combined with metabolic flux analysis may be required.

Our findings may further explain the early lethality of PSS1/2 deficiency in mouse [9, 10]. Defective mitochondrial function or impaired insulin pathway activity could both lead to embryonic lethality. The lethality of the *Drosophila pss* mutant and the pleiotropic phenotypes caused by RNAi of *pss* suggest that dietary/maternally derived PS is either insufficient or cannot be delivered to internal cells/tissues to ensure normal physiological function.

After synthesis, PS can be transported from the ER to the PM or to mitochondria. Little is known about other PS transport routes or how cells coordinate or prioritize the different PS trafficking routes [50]. The genetic suppression of *pss* RNAi by *PI4KIIIα* RNAi indicates that there is a balance between PS transport from the ER to PM and from the ER to mitochondria. In support of this transport balance, the enhancement of the cell growth defect in *pss RNAi* by *Pisd* overexpression can also be explained by diversion of PS away from the ER to PM transport path. Furthermore, the suppression of the mitoEYFP defects of *pss* RNAi by *PI4KIIIα* RNAi opens up a new possibility to screen for more suppressors. The identification and analysis of suppressors will be important for revealing other PS transport routes and their regulation.

## Materials and methods

### *Drosophila* husbandry and stocks

All the flies used in this study (S1 Table) were maintained on standard cornmeal food. The *w*^*1118*^, *ppl-GAL4/+* or *ppl>white RNAi* was chosen as the wild type or control RNAi group. For overexpression, UAS-GMA (GFP tagged actin-binding domain of Moe) or UAS-myr-mRFP (membrane RFP) was used as the UAS control. The *CG4825*^*KK105709*^ line was used in *pss RNAi* experiments if not specified. The fly stocks were obtained from the Bloomington *Drosophila* Stock Center (BDSC), the Vienna *Drosophila* Resource Center (VDRC), NIG Stock Center (NIG) and Tsinghua Fly Center (THFC).

### Tissue staining, microscopy and image analysis

The lipid droplets of wandering 3^rd^ instar larval salivary gland were stained by BODIPY, Nile red, or LipidTOX following the procedure described before [30, 39, 51]. After fixation with 4% paraformaldehyde (PFA) for 0.5 hour, the samples were stained with DAPI (2 ng/μl). For immunofluorescent staining of ATP5A or Akt, the dissected 3^rd^ instar larval salivary glands were fixed in 4% PFA followed by treatment with 0.3% PBST (PBS + 0.3% TritonX-100), and blocked with 5% BSA for 1 hour. The samples were incubated with anti-ATP5A (diluted at 1:200; Abcam, ab14748) or anti-Akt (diluted at 1:200; Cell Signaling) overnight at 4°C. Alexa Fluor 488-conjugated goat anti-mouse (1:1000; Invitrogen) or Alexa Fluor 488-conjugated goat anti-rabbit (1:1000; Invitrogen) was chosen as the secondary antibody, responsively. The stained samples were mounted in 80% glycerol after washing with 1×PBS three times. All the images were acquired by confocal microscopy (Leica SP8) using 20× and 63× objectives, with NA 0.75 and 1.4, respectively. The wavelengths of the laser are 405 nm, 488 nm, 553 nm and 638 nm for DAPI, BODIPY, Nile Red and LipidTOX, respectively. The quantifications of the salivary gland size and cell size were performed by measurement of the cross-sectional area with Image J software (1.51j8).

### Molecular biology and qRT-PCR

For RT-PCR, total mRNAs were isolated from wandering 3^rd^ instar larval salivary glands using Trizol reagent (Invitrogen) and the cDNA was generated using the Superscript II reverse transcriptase kit (Invitrogen). qRT-PCR was performed with the Stratagene MX300P system (Agilent) using Trans Start Green qPCR SuperMix (Transgene Biotech). The expression level of each gene was normalized to *rp49*. The primers used in this study are listed in S2 Table. To make the *UAS-flag-pss* transgene, the coding region of *pss* was cloned from *w*^*1118*^ and inserted into *pUAST-attB-flag* through the *NotI* and *XbaI* sites.

### Phylogenetic analysis

The *Drosophila* PSS sequence was analyzed with Pfam [52]. PSS family members were aligned with the global sequence alignment software ClustalW, and the Neighbor-Joining phylogenetic tree of the PSS family was constructed with MEGA (6.06) [53]. The phylogenetic analysis was conducted with the bootstrap method using 1,000 bootstrap replications.

### NBD-labeled phospholipid chase and TLC

The procedure for the pulse-chase analysis of the S2 cells was modified from Miyata [27] and Tamura [54]. The S2 cells were incubated with NBD-PA, NBD-PC or NBD-PE (Avanti Polar Lipids, Inc.) for 20 min. The S2 cells were washed, then incubated for different periods of time. After harvesting, the total lipids in the cells were extracted and resuspended in chloroform/methanol (1:2, vol/vol). The lipid samples were separated by TLC on silica gel 60 F_254_ plates (Merck,1.05729.0001) using a solvent system of chloroform/methanol/25% ammonia, 65:35:5 [26]. The TLC plates were detected with a Typhoon 9500 imager and the images were analyzed with Image J (1.51j8).

### Cell culture and RNAi in cultured cells

The siRNAs for human PTDSS1 and PTDSS2 (S3 Table) were designed and synthesized by GenePharma Co., Ltd (www.genepharma.com). HeLa cells were cultured in high-glucose DMEM medium (HyClone) supplemented with 10% fetal bovine serum, 100 U/ml penicillin and 100 μg/ml streptomycin (HyClone) at 37°C. The HeLa cells were transfected with 100 pmole of *PTDSS1* or/and *PTDSS2* siRNA and 0.7 pmole mitoEYFP expression plasmid using Lipofectamine 2000 (Invitrogen) for 48 hours.

### Lipidomic analysis

Lipids were extracted form salivary glands of wandering 3^rd^ instar larvae and analyzed as previously described [55]. The samples for each genotype contained 25 pairs of salivary glands. The mole fraction of each lipid was normalized to the mole fraction of total polar lipids.

### Western blot and quantification

The salivary glands were dissected from 60 wandering 3^rd^ instar larvae. The samples were lysed in 240 μl of ice-cold 1% SDS lysis buffer. 10 μg of the sample protein were loaded and detected with the following rabbit antibodies: anti-Akt (Cell Signaling, diluted at 1:1000), anti-phospho-Akt (Ser473) (Cell Signaling, diluted at 1:1000), and rabbit anti-α-tubulin (Abcam, diluted at 1:4000). Quantification of the band intensities was conducted using Image J software (1.51j8) and the protein levels were normalized to tubulin.

### High-pressure freezing (HPF) electron microscopy imaging

The 3rd instar larval salivary gland samples were loaded into carriers and cryofixed on a Leica Microsystems HPM 100 (EM ICE) at ~2,100 bar and automatically cooled into liquid nitrogen. After HPF, the samples were transferred under liquid nitrogen to a Leica Microsystems AFS-2 unit and incubated at -90°C for 72 h in freeze substitution solution: acetone with 2% (wt/vol) osmium tetroxide and 2% (vol/vol) water. The temperature of the samples was gradually increased according to the following timeline: increase by 8°C/h for 4 h; hold at -60°C for 12 h; increase by 5°C/h for 6 h; hold at -30°C for 10 h; increase at 4°C/h for 10 h; hold at 10°C for 10 h. Samples were washed four times in acetone, stained in 1% uranyl acetate for 1 h, and rinsed 3 times in pure acetone. Samples were infiltrated stepwise with increasing concentrations of Embed 812 resin: 2:1 (Embed 812:acetone) for 3 h, 1:1 for 5 h, then twice in 100% fresh resin for 8 h. The samples were then transferred to an embedding mold containing fresh resin and polymerized in a 60°C oven for 3 days. Ultrathin sections (60 nm) were produced with a diamond knife (Diatome) on an ultramicrotome (Ultracut UCT; Leica Microsystems). The sections were all collected on slot copper grids (EMS), then visualized with a JEM 1400 TEM (Hitachi 7700) operating at 80 kV. Pictures were recorded with a Gatan 832 4kX2.7k CCD camera.

### Statistical analysis

All the data are shown as mean ± SEM. All the statistical analyses were conducted with R language (3.5.1) and R packages (Rcmdr). The graphs were drawn by GraphPad (version 7.00).

## Supporting information

S1 Fig *pss RNAi* reduces cell size. (A) Quantification of the cell number in *ppl-GAL4/+*, *ppl>control RNAi* and *pss RNAi* 3^rd^ instar larval salivary glands (n = 7). The salivary gland cell number is not changed when *pss* is knocked down.
(B) Quantification of the cell size in *ppl-GAL4/+*, *ppl>control RNAi* and *pss RNAi* 3^rd^ instar larval salivary glands (n = 5). The salivary gland cell size is reduced in *pss RNAi* compared to *ppl-GAL4/+*.
(C) Lipid droplet staining in 3^rd^ instar larval salivary glands from four *CG4825* RNAi lines. Scale bar represents 100 μm. BODIPY (green) labels lipid droplets and DAPI (blue) labels nuclei.
(A and B) Data are shown as mean ± SEM. Data were compared with One-way ANOVA. *** p < 0.001.
(TIF)

S2 Fig Genetic analysis of *pss* and *Pisd*. (A) The transcription level of Akt in 3^rd^ instar larval salivary glands with *pss RNAi* (n = 3, each repeat contains RNA from 25 larvae). Data are shown as mean ± SEM. Data were compared with the unpaired Welch Two Sample *t*-test.
(B) Over-expression of PI3K^CA^ in *pss RNAi* suppresses the reduced salivary gland size and but not ectopic lipid accumulation phenotypes. BODIPY (green) labels lipid droplets and DAPI (blue) labels nuclei. Scale bar represents 100 μm.
(C) RNAi of *Pisd* in *pss RNAi* does not suppress the reduced salivary gland size and ectopic lipid accumulation phenotypes. Nile Red (red) labels lipid droplets and DAPI (blue) labels nuclei. Scale bar represents 100 μm.
(D) The RNAi knockdown efficiency of *Pisd* (n = 3, each repeat contains RNA from 25 larvae). Data are shown as mean ± SEM. Data were compared with the unpaired Welch Two Sample *t*-test. *p < 0.*05*.
(TIF)

S3 Fig *pss RNAi* impairs mitochondrial protein import. (A) Images of MitoTimer-labeled mitochondria in *ppl-Gal4/+*, *ppl>control RNAi* and *pss RNAi* 3^rd^ instar larval salivary gland. MitoTimer is detected in two forms: GFP and dsRed. Scale bar represents 20 μm.
(B) The transcription level of mitoEYFP in 3^rd^ instar larval salivary glands with *pss RNAi* (n = 3, each repeat contains RNA from 25 larvae). Data are shown as mean ± SEM. Data were compared with the unpaired Welch Two Sample *t*-test.
(C) *pss RNAi* does not affect the expression of other GFP/myr-mRFP reporters. Scale bar represents 50 μm.
(D) The crista junction width in *pss RNAi* cell mitochondria is increased. Scale bar represents 0.2 μm.
(TIF)

S1 Table The fly strains used in this study. (DOCX)

S2 Table The primers used in this study. (DOCX)

S3 Table The siRNAs used in this study. (DOCX)
