# Supplementary figures and images for: Phosphatidylserine synthase regulates cellular homeostasis through distinct metabolic mechanisms
Source: PLoS Genet. 2019 Dec 23;15(12):e1008548. doi: 10.1371/journal.pgen.1008548 (PMC6946173; doi:10.1371/journal.pgen.1008548)

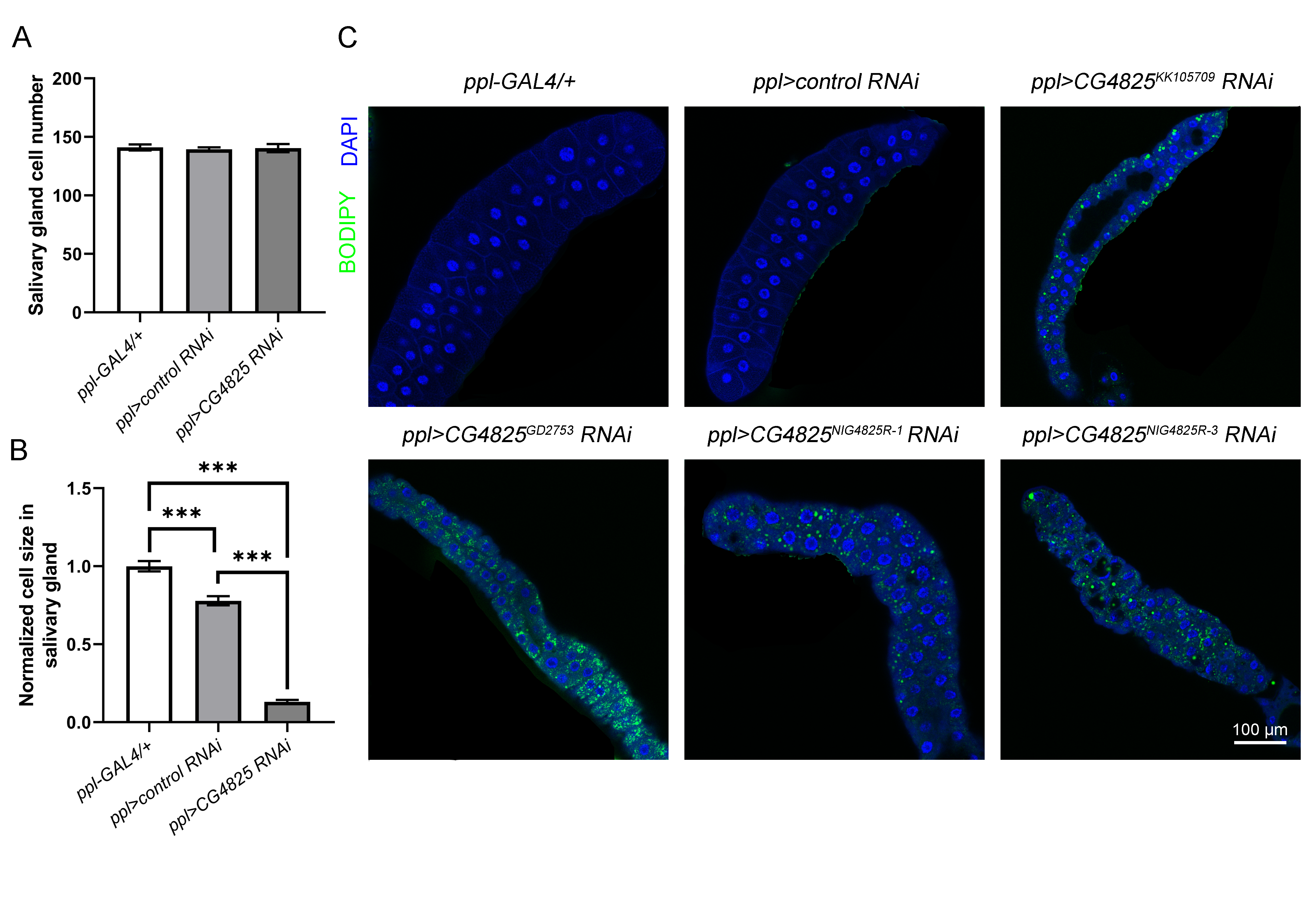

Supplement: S1 Fig — (A) Quantification of the cell number in ppl-GAL4/+, ppl>control RNAi and pss RNAi 3rd instar larval salivary glands (n = 7). The salivary gland cell number is not changed when pss is knocked down. (B) Quantification of the cell size in ppl-GAL4/+, ppl>control RNAi and pss RNAi 3rd instar larval salivary glands (n = 5). The salivary gland cell size is reduced in pss RNAi compared to ppl-GAL4/+. (C) Lipid droplet staining in 3rd instar larval salivary glands from four CG4825 RNAi lines. Scale bar represents 100 μm. BODIPY (green) labels lipid droplets and DAPI (blue) labels nuclei. (A and B) Data are shown as mean ± SEM. Data were compared with One-way ANOVA. *** p < 0.001. (TIF) [file pgen.1008548.s001.tif]

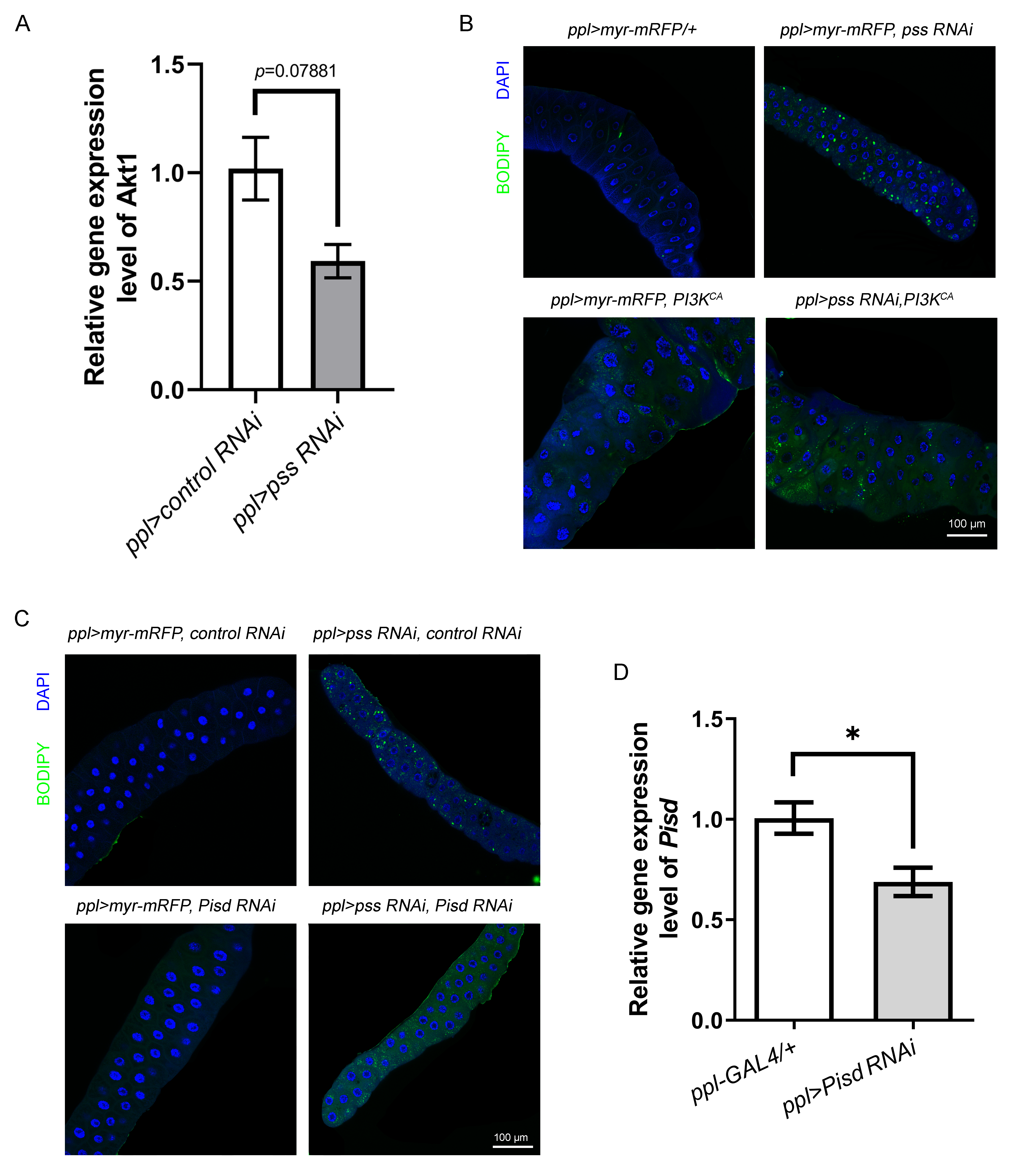

Supplement: S2 Fig — (A) The transcription level of Akt in 3rd instar larval salivary glands with pss RNAi (n = 3, each repeat contains RNA from 25 larvae). Data are shown as mean ± SEM. Data were compared with the unpaired Welch Two Sample t-test. (B) Over-expression of PI3KCA in pss RNAi suppresses the reduced salivary gland size and but not ectopic lipid accumulation phenotypes. BODIPY (green) labels lipid droplets and DAPI (blue) labels nuclei. Scale bar represents 100 μm. (C) RNAi of Pisd in pss RNAi does not suppress the reduced salivary gland size and ectopic lipid accumulation phenotypes. Nile Red (red) labels lipid droplets and DAPI (blue) labels nuclei. Scale bar represents 100 μm. (D) The RNAi knockdown efficiency of Pisd (n = 3, each repeat contains RNA from 25 larvae). Data are shown as mean ± SEM. Data were compared with the unpaired Welch Two Sample t-test. *p < 0.05. (TIF) [file pgen.1008548.s002.tif]

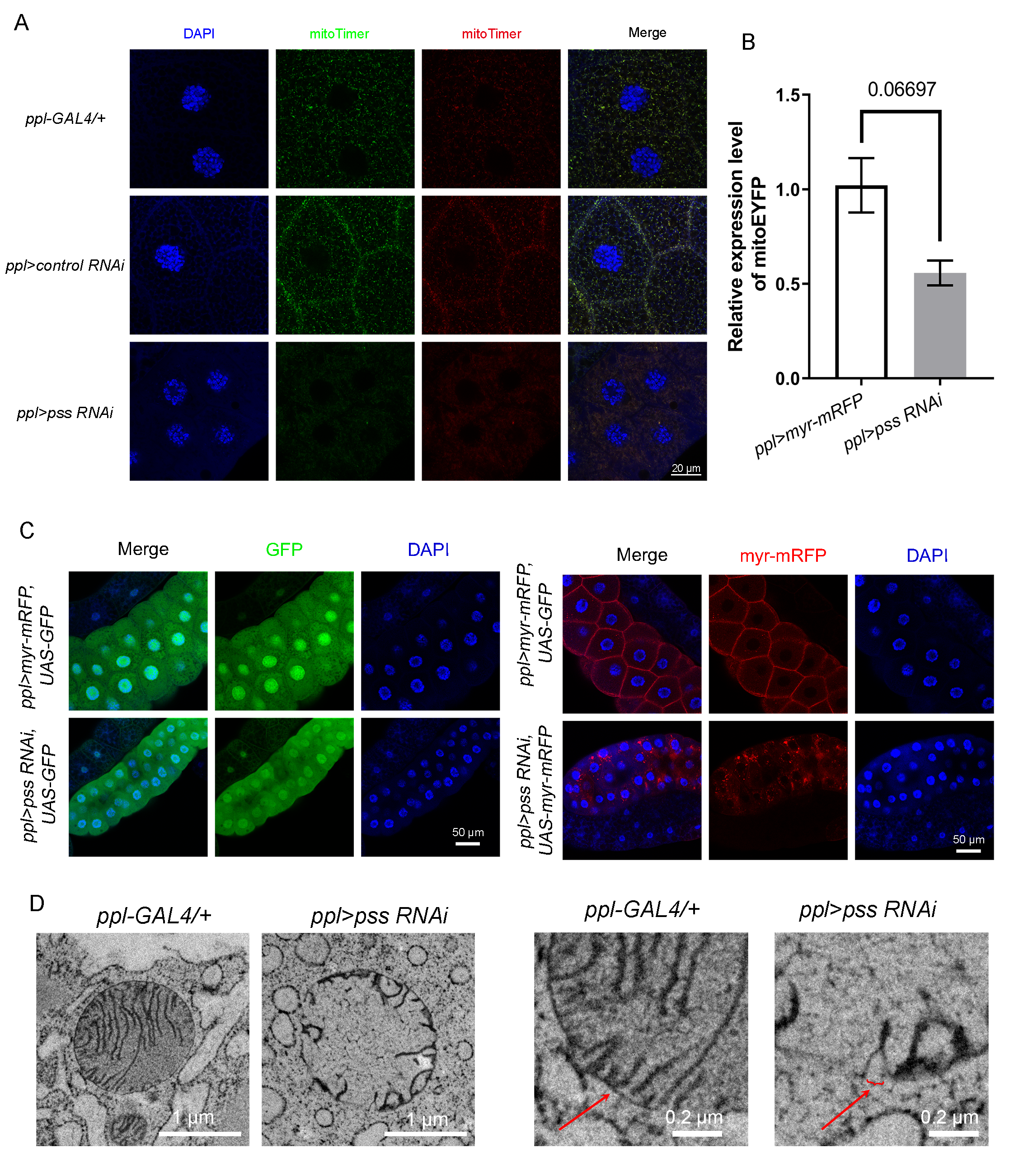

Supplement: S3 Fig — (A) Images of MitoTimer-labeled mitochondria in ppl-Gal4/+, ppl>control RNAi and pss RNAi 3rd instar larval salivary gland. MitoTimer is detected in two forms: GFP and dsRed. Scale bar represents 20 μm. (B) The transcription level of mitoEYFP in 3rd instar larval salivary glands with pss RNAi (n = 3, each repeat contains RNA from 25 larvae). Data are shown as mean ± SEM. Data were compared with the unpaired Welch Two Sample t-test. (C) pss RNAi does not affect the expression of other GFP/myr-mRFP reporters. Scale bar represents 50 μm. (D) The crista junction width in pss RNAi cell mitochondria is increased. Scale bar represents 0.2 μm. (TIF) [file pgen.1008548.s003.tif]
