# Supplementary material for: Phosphatidylserine synthase regulates cellular homeostasis through distinct metabolic mechanisms
Source: PLoS Genet. 2019 Dec 23;15(12):e1008548. doi: 10.1371/journal.pgen.1008548 (PMC6946173; doi:10.1371/journal.pgen.1008548)
Supplement: S1 Table — (DOCX) [file pgen.1008548.s004.docx]

**Supplementary Table S1. The fly strains used in this study.**

| **ID** | **Description** | **Genotype** | **Source** |
| --- | --- | --- | --- |
| THU0583 | *w RNAi* | *P{TRiP.HMS00045}attP2* | THFC |
| v105470 | *pss* (*CG4825*) *RNAi* | *P{KK105709}VIE-260B* | VDRC |
| v5391 | *pss* (*CG4825*) *RNAi* | *w^1118^; P{GD2753}v5391/CyO* | VDRC |
| NIG4825R-1 | *pss* (*CG4825*) *RNAi* | *CG4825^NIG.4825R-1^* | NIG |
| NIG4825R-3 | *pss* (*CG4825*) *RNAi* | *CG4825^NIG.4825R-3^* | NIG |
| BL42044 | *pss* (*CG4825*) mutant | *y^1^ w^*^; Mi{MIC}CG4825^MI01234^/TM3, Sb^1^ Ser^1^* | BDSC |
| BL14017 | *pss* (*CG4825*) mutant | *y^1^; P{SUPor-P}CG4825^KG06018^ ry^506^/TM3, Sb^1^ Ser^1^* | BDSC |
| NIG5991R-2 | *Pisd RNAi* | *Pisd^NIG.5991R-2^* | NIG |
| BL15442 | *Pisd* overexpression | *y^1^ w^67c23^; P{EPgy2}Pisd^EY03559^* | BDSC |
| THU2146 | *Tom40 RNAi* | *y^1^ v^1^; P{TRiP.JF02030}attP2* | THFC |
| BL16893 | *CdsA* overexpression | *y^1^ w^67c23^; P{EPgy2}Cds^EY08412^* | BDSC |
| v36006 | *Lipin RNAi* | *w^1118^; P{GD14004}v36006* | VDRC |
| THU3758 | *PI4KIIIα RNAi* | *y^1^ sc* v^1^ sev^21^;P{TRiP.HMS01686}attP40* | THFC |
| BL25908 | *PI3K^CA^* | *P{Dp110-CAAX}1, y^1^ w^*^* | BDSC |
| BL50758 | *Akt^CA^* | *w^*^; P{UAS-myr-Akt1.V}3/TM3, Sb^1^* | BDSC |
| BL8164 | *tGPH* | *w^1118^; P{tGPH}4* | BDSC |
| BL7194 | *mitoEYFP* | *w^*^; P{sqh-EYFP-Mito}3* | BDSC |
| BL53752 | *MitoTimer* | *w^1118^; P{UAS-MitoTimer}3* | BDSC |
|  | *ppl-GAL4* driver | *w^*^; P{ppl-GAL4.P}2* | Pierre Léopold |
| BL1824 | *AB1-GAL4* driver | *y^1^ w^*^; P{GawB}AB1* | BDSC |
| BL31776 | *UAS-GMA* | *w^1118^; P{UAS-GMA}3* | BDSC |
|  | *UAS-GFP* | *UAS-GFP* | Huang Lab |
| BL7118 | *UAS-myr-mRFP* | *w^1118^; P{UAS-myr-mRFP}1* | Huang Lab |
|  | *Flag-PSS* over-expression | *UAS-flag-pss* | Huang Lab |

Resource sharing information and contact:

Xun Huang ([xhuang@genetics.ac.cn](mailto:xhuang@genetics.ac.cn)).

Annotation:

1. *AB1-GAL4*: Expresses GAL4 in salivary gland (basal expression of P{GawB}).
2. *UAS-GMA*: Expresses the actin-binding domain of moesin tagged with GFP[S65T] under the control of UAS.
3. *UAS-mRFP* (*UAS-myr-mRFP*): Expresses a membrane-targeted monomeric RFP under the control of UAS.
