# Supplementary material for: Phosphatidylserine synthase regulates cellular homeostasis through distinct metabolic mechanisms
Source: PLoS Genet. 2019 Dec 23;15(12):e1008548. doi: 10.1371/journal.pgen.1008548 (PMC6946173; doi:10.1371/journal.pgen.1008548)
Supplement: S2 Table — (DOCX) [file pgen.1008548.s005.docx]

**Supplementary Table S2. The primers used in this study.**

| **Primer name** | **Primer sequence (5'-3')** | **Usage** |
| --- | --- | --- |
| CG4825-qFw1 | ACTGTCCAAGTAATAATCTCGGTG | qRT-PCR |
| CG4825-qRv1 | GGCGACAATTTACCTTTCGAG | qRT-PCR |
| Dmel-Akt1-qFw1 | CATGTACGAGATGATCTGTGG | qRT-PCR |
| Dmel-Akt1-qRv1 | AGAATGGATGTGCTTGTATCTC | qRT-PCR |
| rp49-qFw1 | CCCAACCTGCTTCAAGATGAC | qRT-PCR |
| rp49-qRv1 | CGCACTCTGTTGTCGATACC | qRT-PCR |
| PTDSS1-qFw | GCAAGTGGAGGACATCACCAT | qRT-PCR |
| PTDSS1-qRv | TCATCCCTGGTAAAGGCGAAG | qRT-PCR |
| PTDSS2-qFw | CTCACCTGTACGCTTGGCTAT | qRT-PCR |
| PTDSS2-qRv | CCACAATACCTCTCTTGGTGTTG | qRT-PCR |
| GAPDH-qFW | GGAGCGAGATCCCTCCAAAAT | qRT-PCR |
| GAPDH-qRv | GGCTGTTGTCATACTTCTCATGG | qRT-PCR |
| Pisd-qFw1 | AATAGTCTGGCTGAGTTCTTTACC | qRT-PCR |
| Pisd-qRv1 | CTAACACCCTTGACCTGCTC | qRT-PCR |
| mitoEYFP-qFw | GCACCATCTTCTTCAAGGAC | qRT-PCR |
| mitoEYFP-qRv | GGCTGTTGTAGTTGTACTCC | qRT-PCR |
| NotI-pss-Fw | ATAAGAATGCGGCCGCATGAAGAAGCGCACTAATTCACG | transgene |
| XbaI-pss-Rv | GCTCTAGATTATTTTAGTTTCTTCTCTTTTTGCGGC | transgene |
