# Supplementary material for: Phosphatidylserine synthase regulates cellular homeostasis through distinct metabolic mechanisms
Source: PLoS Genet. 2019 Dec 23;15(12):e1008548. doi: 10.1371/journal.pgen.1008548 (PMC6946173; doi:10.1371/journal.pgen.1008548)
Supplement: S3 Table — (DOCX) [file pgen.1008548.s006.docx]

**Supplementary Table S3. The siRNAs used in this study.**

| **siRNA** | **Sense (5'-3')** | **Anti-sense (5'-3')** |
| --- | --- | --- |
| Negative control | UUCUCCGAACGUGUCACGUTT | ACGUGACACGUUCGGAGAATT |
| GAPDH | UGACCUCAACUACAUGGUUTT | AACCAUGUAGUUGAGGUCATT |
| PTDSS1 | GCAGCUGACUGAGUUGAAUTT | AUUCAACUCAGUCAGCUGCTT |
| PTDSS2 | GCACCGAGUCCGAGGUCUATT | UAGACCUCGGACUCGGUGCTT |
